# Cocooning strategy: Pertussis vaccination coverage rate of parents with a new-born in 2016 and 2017 in France

**DOI:** 10.3389/fped.2022.988674

**Published:** 2022-10-18

**Authors:** Clarisse Marchal, Manon Belhassen, Nicole Guiso, Flore Jacoud, Robert Cohen, Marie Le Pannerer, Régis Verdier

**Affiliations:** ^1^PELyon (Pharmaco Epidemiology Lyon), Lyon, France; ^2^Independent Expert, Paris, France; ^3^Université Paris Est, IMRB-GRC GEMINI, Créteil, France; ^4^Clinical Research Center, Centre Hospitalier Intercommunal de Créteil, Créteil, France; ^5^GPIP, Pediatric Infectious Disease Group, Créteil, France; ^6^ACTIV, Pediatric Clinical and Therapeutical Association of the Val de Marne, Saint-Maur-des-Fossés, France; ^7^Unité Court Séjour, Petits Nourrissons, Service de Néonatologie, Centre Hospitalier Intercommunal de Créteil, Créteil, France; ^8^Sanofi Pasteur, Medical Affairs, Lyon, France

**Keywords:** vaccination coverage rates, claims database, pertussis, cocooning strategy, new parents

## Abstract

**Background:**

The “cocooning” strategy was introduced in 2004 to protect infants too young to be vaccinated against pertussis, by immunizing their parents and close relatives. The study objective was to assess its implementation 12 years after its introduction by estimating the pertussis vaccination coverage rates (VCR) among parents of newborns.

**Materials and methods:**

Pertussis VCR were estimated among all women who gave birth and men who took paternity leave, in 2016 or 2017, from a 1/97th random sample of French claims data. Two distinct study periods were defined based on current recommendations for the cocooning strategy: the “common practice” and the “parental project” periods.

**Results:**

In 2016, the pertussis VCR of women having given birth and men having taken paternity leave was 47.2 and 47.1%, respectively (46.1 and 45.6% in 2017, respectively). About one quarter of vaccinations were performed during the “parental project” period, with the vaccine most frequently reimbursed during the month of childbirth for women (57.1% in 2016 and 49.4% in 2017) and before or during the month the paternity leave began for men (about 78% in both 2016 and 2017). General practitioners were the main prescribers in private practice, even during the “parental project” period.

**Conclusion:**

To optimize the protection for infants, the main objective of the cocooning strategy, pertussis immunization coverage of adults and seniors needs to be improved. Moreover, cocooning vaccination linked to a parental project needs to be performed earlier, during pregnancy (for those around the mother) or in immediate *post-partum* (e.g., during the maternity stay).

## Introduction

Pertussis is a highly contagious bacterial infection of the respiratory tract potentially severe at any age. This disease is particularly dangerous, even life-threatening, in non-vaccinated infants under 6 months of age and at-risk persons such as pregnant women and elderly people (1).

Globally, it was estimated that there were 24.1 million pertussis cases and 160,700 deaths from pertussis in children less than 5 years of age in the world in 2014 (2). In France, the incidence rate of pertussis was 290 per 100,000 infants aged 0–2 months in 2012 (3) and is still one of the first causes of death from bacterial infection in infants under 3 months of age (4).

In France, the vaccination of children against pertussis is mandatory from the age of 2 months since January 1, 2018. Since the immunization schedule published in 2013, primary vaccination schedule consists of two injections 2 months apart, followed by a booster at 11 months (5). Moreover, pertussis booster vaccination is now recommended at 6 years of age, in combination with Diphtheria, Tetanus and Poliomyelitis vaccination (DTaP-IPV) and at 11–13 years and 25 years of age with a reduced-dose vaccine in diphtheria and pertussis antigens (Tdap-IPV). Since 2004, this strategy is completed by the “cocooning” strategy: it aims to protect infants too young to be vaccinated against pertussis, by immunizing their parents and close relatives (6). The strategy targets primarily adults planning on having a child. For parents who have a parental project, the childbearing mother should be vaccinated prior to being pregnant, or just after the birth, and any person likely to be in close contact with the newborn during the first 6 months of his life (children, partner, etc.) should also be vaccinated, ideally before the birth. A study conducted in France 10 years after the implementation of the cocooning strategy (Vaccinoscopie^®^) demonstrated that 61% of mothers and 42% of fathers of infants aged < 12 months were adequately immunized against pertussis (7).

The French-universal healthcare coverage and its unified national healthcare data system (8) represents a relevant tool to estimate pertussis vaccination coverage among new parents, thus assessing the implementation of the cocooning strategy 12 years after its introduction. Our study aimed at estimating the pertussis vaccination rate among women who gave birth and men who have taken paternity leave in 2016 and 2017 in France. We also describe the characteristics of the healthcare professional (HCP) who prescribed the pertussis vaccination.

## Materials and methods

### Study design and data source

This was a historical cohort study conducted within the French General Sample of Beneficiaries (EGB), which is a 1/97e random sample of the French health insurance reimbursement database. The EGB records individual anonymous information from primary and secondary care (data from PMSI, the French Diagnosis Related Group-based medical information system), and it currently covers more than 80% of the French population. It contains: (a) characteristics (gender, month and year of birth, month and year of death if applicable); (b) all non-hospital reimbursed healthcare expenditures with date and code (visits and medical procedures, laboratory tests, drugs, and medical devices, but not the corresponding medical indication or results); (c) hospital discharge summaries (ICD-10 diagnoses codes for all medical, obstetric, and surgery hospitalizations with the date and duration of hospitalization, medical procedures, hospital department, and cost coding system) (8, 9). For this study, we extracted the general characteristics, date and code of non-hospital reimbursed healthcare expenditures for DTaP-IPV and Tdap-IPV, as well as the hospital discharge and medical procedure codes for childbirth.

### Study population

We enrolled all women who gave birth and men who took paternity leave in 2016 or 2017, with enough historical data, i.e., available data over the following periods:

(i) In the 10 years before (5 years for women 25 years-old or under) and in the 6 months after childbirth for women,
(ii) In the 10 years before (5 years before for men 25 years-old or under) and in the 5 months after the start of paternity leave for men.

We used these time periods to track back the immunization status of the enrolled population. This is in line with the current recommendations for pertussis vaccination within the cocooning strategy (10).

As it is not possible to directly find fathers corresponding to each childbirth in the EGB, we identified men with a newborn using the start date of their paternity leave. We identified childbirth using the algorithm developed by the French National Health Insurance Fund and the French Health Agency (11, 12).

### Variables

For each person, we identified the following variables within the database: age at childbirth, characteristics of the HCP who prescribed the pertussis vaccine (private or hospital setting and prescriber specialty in private practice), and pertussis vaccination status.

In women, the pertussis Vaccination Coverage Rate (VCR) was estimated as the ratio of women having received a shot of vaccine containing pertussis valence (DTaP-IPV or Tdap-IPV), over the tracking periods defined before, to the number of women enrolled, i.e., the number of women having given birth in 2016 or 2017.

Similarly, in men, the VCR was estimated as the ratio of men having received a shot of vaccine containing pertussis valence (DTaP-IPV or Tdap-IPV), over the tracking periods defined before, to the number of men enrolled, i.e., the number of men having taken paternity leave in 2016 or 2017.

### Statistical analysis

We defined two distinct study periods: (1) the “common practice”; i.e., from 10 years before childbirth in parents 25 years or older (5 years before for those under 25) to 9 months before childbirth for women and to 10 months before the start of paternity leave for men; (2) the “parental project” period: from 9 months before to 6 months after childbirth for women, and from 10 months before to 5 months after the start of paternity leave for men. For men, this is based on the assumption that paternity leave would start 1 month after the childbirth as there are no possibilities to track the effective childbirth date. Over each of these two study periods, separately in men and women, age distribution (mean, standard deviation, min-max), the pertussis VCR in percentage, timing for vaccination (i.e., distribution of the percentage of vaccination according to the month of the “parental project” period) and characteristics of HCP (proportion of prescriptions from private versus hospital setting and proportion of each prescriber specialty in private practice) were described. The cumulative percentage of vaccinated persons were estimated by month over the “parental project” period for persons vaccinated during this period.

### Regulatory aspects

This observational study was conducted on anonymized data and the National Informatics and Liberty Committee has delivered an overall authorization to use EGB data for research purposes. This study was performed after approval by the Health Data Hub, approval n° 2856825.

## Results

### Person’s characteristics

**FIGURE 1:**
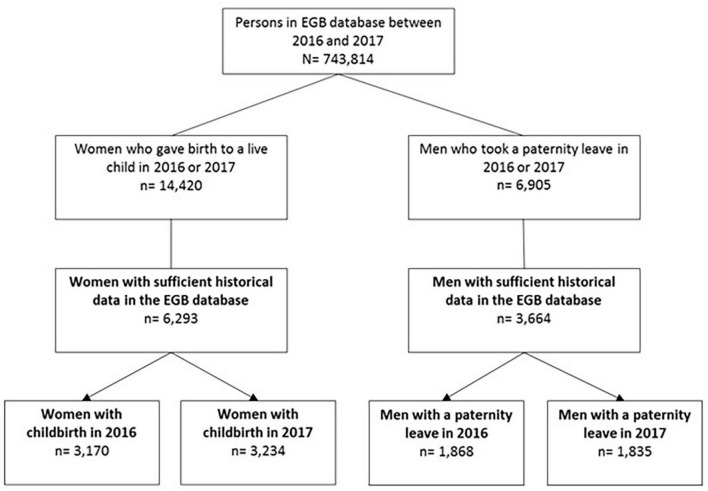
Flowchart.

Of the 743,814 persons in the EGB database, we included 3,170 and 3,234 women who gave birth to a live child and 1,868 and 1,835 men who took paternity leave in 2016 and 2017, respectively, with enough historical data in the EGB (Figure 1).

The mean age of selected women who were vaccinated was 31 years old, slightly higher than those who were not vaccinated (29 years old). The mean age of selected men was similar in vaccinated and in unvaccinated men, i.e., 34 years old. Mean ages were similar in 2016 and in 2017 for both women and men (Table 1).

**TABLE 1:** Age of women and men according to their vaccination status and the year of childbirth/beginning of paternity leave.

Gender	2016			2017		
		
	*N* (%)	Mean age (SD)	Min–max age	*N* (%)	Mean age (SD)	Min–max age
**Women**						
Unvaccinated	1,673 (52.8%)	29.3 (6.5)	16.0–49.0	1,742 (53.9%)	29.6 (6.5)	14.0–48.0
Vaccinated	1,497 (47.2%)	31.0 (5.9)	15.0–50.0	1.492 (46.1%)	31.2 (5.8)	14.0–49.0
**Men**						
Unvaccinated	988 (52.9%)	34.1 (6.4)	19.0–62.0	998 (54.4%)	34.2 (6.6)	19.0–63.0
Vaccinated	880 (47.1%)	34.4 (5.7)	21.0–57.0	837 (45.6%)	34.3 (6.2)	19.0–58.0

### Pertussis vaccination coverage rate and time of vaccination

**FIGURE 2:**
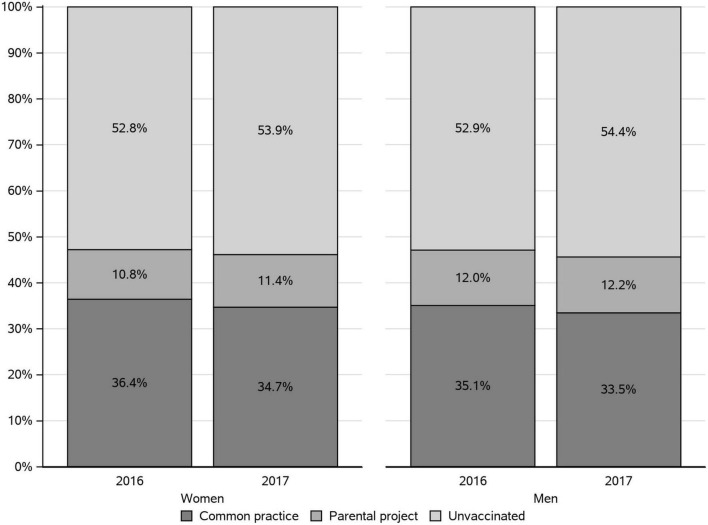
Pertussis vaccination rate of women and men parents in 2016 and 2017, by period in which vaccine reimbursement was recorded.

In 2016, the pertussis VCR of women having given birth and men having taken paternity leave was 47.2 and 47.1%, respectively (Figure 2). In 2017, the pertussis VCR were similar (46.1 and 45.6% of women and men, respectively). Both in men and women, most vaccinations (about three quarters) were performed during the “common practice” period and about one quarter of the vaccinations were performed during the “parental project” period.

**FIGURE 3:**
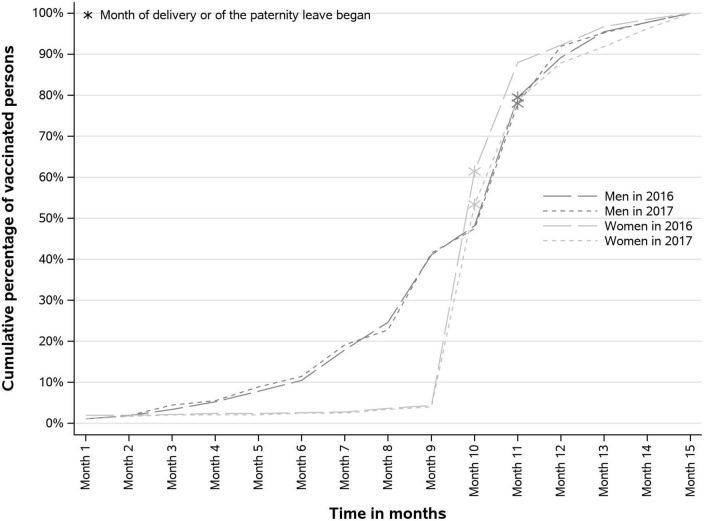
Cumulative vaccination coverage rates (VCR) during the “parental project” period, according to the month of the “parental project.”

In women who were vaccinated during the “parental project” period, the vaccine was most frequently reimbursed during the month of the childbirth (57.1% in 2016 and 49.4% in 2017). Two months after childbirth, more than three quarters of “parental project” vaccination were performed (Figure 3). About half of the men vaccinated (48%) during the “parental project” were vaccinated before the month the paternity leave began, and about 30% were vaccinated during that month.

### Characteristics of the healthcare professional prescribing the pertussis vaccination

**FIGURE 4:**
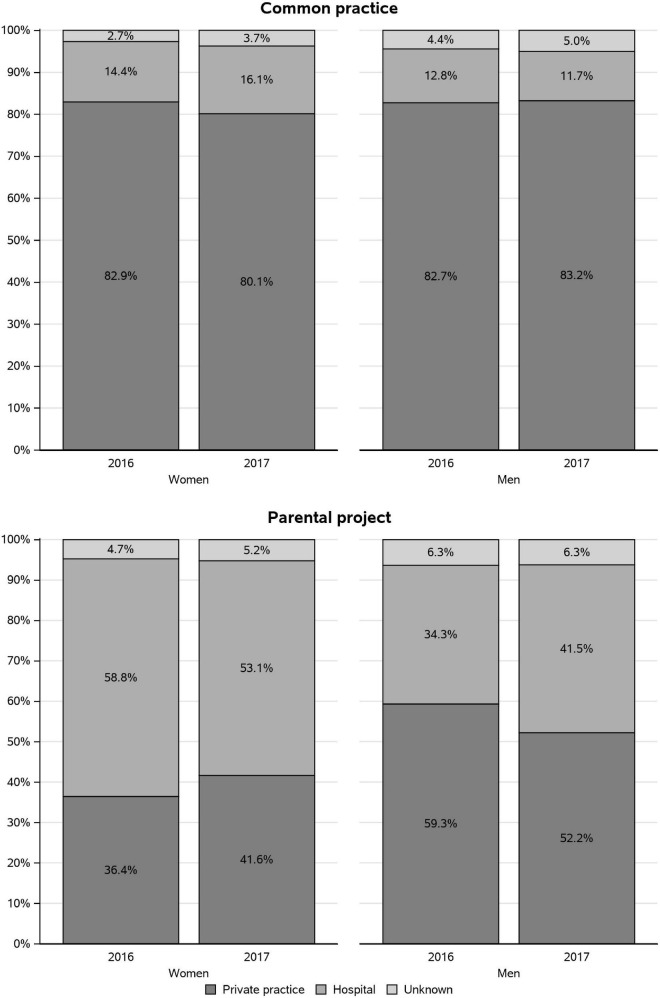
Private or hospital practice setting of the healthcare professional (HCP) who prescribed the pertussis vaccine in 2016 and 2017, according to the study period.

Most of the vaccinations occurring during the “common practice” period were initiated in private practice (more than 80%, both in women and in men) (Figure 4). The vaccinations occurring during the “parental project” period were also mostly initiated in private practice in men (59.3 and 52.2% in 2016 and 2017, respectively). In contrast, they were mainly initiated at hospital in women (58.8 and 53.1% in 2016 and 2017, respectively). We observe that vaccination initiated at hospital during the “parental project” increased in men between 2016 and 2017, whereas it decreased in women.

**FIGURE 5:**
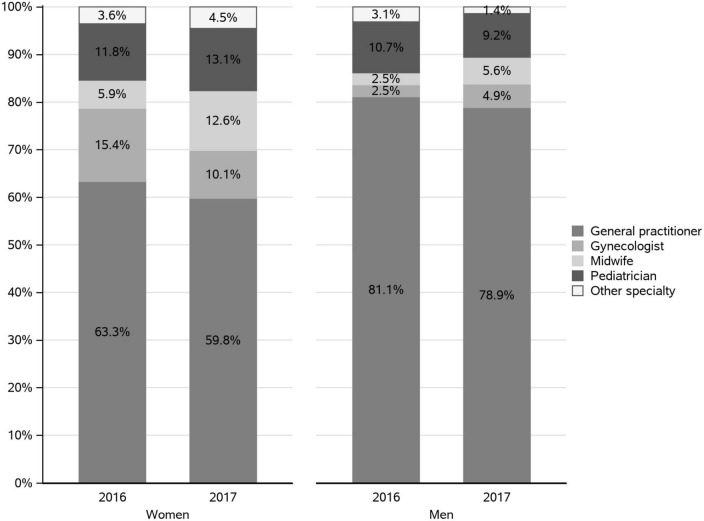
Specialty of the healthcare professional (HCP) who prescribed the pertussis vaccine in private practice during the “parental project” in 2016 and 2017.

During the “common practice” period, the majority (around 93% in both men and women) of the prescriptions of pertussis vaccinations prescribed in private practice were obtained from general practitioners (GPs). During the “parental project” period, the most frequent prescribers of the vaccine in private practice were also the GPs but in lower proportion than during the “common practice” period (63.3 and 59.8% in women and 81.1 and 78.9% in men, in 2016 and 2017, respectively) (Figure 5). In women in 2017, we observed an increase in the prescriptions by midwives (from 5.9 to 12.6%) and a decrease in the prescriptions by gynecologists (from 15.4 to 10.1%). Pediatricians appear as frequent prescribers in the “parental project” period (11.8 and 13.1% in 2016 and 2017).

## Discussion

### Main findings

In 2016 and 2017, less than half of the parents of newborns were vaccinated against pertussis according to the vaccination recommendation. Cocooning vaccination against pertussis during the “parental project” period remained low but appeared to be performed quickly after birth.

During the “parental project”, prescriptions were mainly initiated at hospital for women. General practitioners are the main prescribers in private practice, even during the “parental project” period.

### Internal and external validity

The vaccination periods have been defined according to the cocooning strategy, whose recommendations were not modified over the study period.

The Vaccinoscopie^®^ study, an annual survey in France, revealed, 10 years after the introduction of the cocooning strategy, that 61% of mothers (2014) and 42% of fathers (2013) of infants aged < 12 months were adequately immunized against pertussis (7). The percentage of vaccinated mothers is higher than those observed in our study (47.2 and 46.1% in 2016 and 2017, respectively), probably because the VCR were estimated until the sixth month after childbirth in this study whereas they were estimated in parents of infants aged < 12 months in the Vaccinoscopie^®^ study. Hence, it would be of interest to repeat these analyses in subsequent years to assess whether vaccination rates continue to evolve or whether a plateau is reached.

In a national perinatal survey in France in 2016, 37% of mothers received a booster pertussis vaccination in the 10 years before pregnancy, which is consistent with the percentage observed in our study (36.4% in 2016 and 34.7% in 2017) (13). The same trend was observed in the Vaccinoscopie^®^ study (30% of vaccination between a year before pregnancy and 4 months after giving birth).

The cocooning strategy is recommended to protect newborns during the first 6 months of their lives (when they start benefiting from the direct protection of their own primary vaccination at 2 and 4-month). It is recommended for women to be vaccinated just after childbirth if not vaccinated before pregnancy. Therefore, although for mothers most vaccination during the “parental project” period is observed in the month of childbirth, 38.5% appear to occur after. In the same way, to ensure maximum protection, the cocooning strategy recommends vaccinating the father (and other close contacts) during pregnancy, which is in line with what was observed in this study (almost 50% of “parental project” vaccination in fathers seemed to be performed during pregnancy and around 30% during the month of childbirth). Additionally, as close contacts to the newborn, not only parents but also grand parents can transmit the disease to infants. Although not recommended in France, pertussis immunization for adults would reinforce the herd immunity and bring benefits for direct protection against pertussis, especially for seniors as the disease is more severe and can be dramatic for people over 70-year-old without a booster vaccination. Indeed, the study of Berbers et al showed that *Bordetella pertussis* is also circulating among people over 50 in France (14), in a context of low vaccination coverage rates against pertussis as highlighted by the study of Marchal et al. (15).

The cocooning strategy therefore appears to be partially effective because of the difficulties in ensuring vaccination coverage of all those around the newborn. Moreover, pertussis vaccines protect against the disease but would not completely prevent carriage. The best strategy to protect newborns from pertussis is to make sure they have their own antibodies at birth. Hence, vaccination during pregnancy could reduce the risk of pertussis in newborns and infants through the transplacental transfer of anti-pertussis antibodies and by protecting the mother against this disease. Very few reimbursements during pregnancy were observed in this study. Indeed, vaccination of women during pregnancy was not recommended in France over the study period, although not contraindicated and even recommended by the World Health Organization (WHO) and adopted by several countries (16–18). As this strategy has recently been recommended by the French National Academy of Medicine (19) and the French National Authority of Health (20), an increase in VCR during pregnancy leading to better results in terms of prevention of pertussis in newborns could be expected from 2022.

The high percentage of prescriptions from the hospital in the “parental project” may correspond to prescriptions at discharge from the maternity hospital. Indeed, the High Council of Public Health (*Haut Conseil de Santé Publique*) and the National College of French Gynecologists and Obstetricians (CNGOF) recommend performing the vaccination against pertussis in the immediate postpartum for unvaccinated women (10, 21). Several studies highlighted a positive impact of different interventions in maternity units in France to promote vaccination against pertussis (22–25).

General practitioners also appear to play a key role in cocooning vaccination, as the most frequent prescribers of pertussis vaccines for mothers and fathers in private practice. This is consistent with the results of the study of Cohen et al. (7) in which the GP was the most frequent HCP (57%) to have informed the mothers of the importance of pertussis vaccination during their last pregnancy. Moreover, according to another French study carried out in 2002, 61% of visits for children under the age of one are carried out by a GP, giving them the opportunity to check the mother’s immunization status (26). Even if an increase of prescriptions from midwives in private practice during the “parental project” was observed between 2016 and 2017, the percentage is still low (around 13%). However, this can be explained by the fact that most midwives work in hospitals with no possibility in the database to track the specialty of the prescribers in a hospital setting (27, 28). In contrast, gynecologists work mainly in private practice (29), thus the percentages of prescription coming from them during the “parental project” (15.4% in 2016 and 10.1% in 2017) observed in this study are lower than expected.

### Limitations

Some limitations should be acknowledged. As always the case in claims data, the real use of vaccines remains unknown. We know that the person got the vaccine at the pharmacy, however, it is difficult to deduce whether the vaccine was actually injected from the data obtained. The vaccination coverage may thus have been slightly overestimated, but we can reasonably assume that this overestimation is limited, even if there is no data to estimate the frequency of this situation. Nevertheless, claims data are closer to real consumption than prescriptions data. In order to estimate the VCR in men according to the cocooning strategy, we assumed that the paternity leave started 1 month after birth, but in practice, it may begin up to 4 months after childbirth. Consequently, we cannot ascertain that the vaccinations in men carried out from the beginning of paternity leave occurred right after the childbirth which is the essence of the cocooning strategy to best protect the infant. Moreover, we selected men who took and declared a paternity leave, thus who are not representative of all fathers-to-be. Indeed, the selected fathers may be more involved in the care of the child and therefore more likely to be vaccinated.

In order to assess the vaccination coverage, we selected only the population with enough historical data. In people over 25 years old, only those affiliated to the general health insurance scheme could have 10 years of historical data and be included, due to variations in the population of the EGB. People under 25 years, for whom only 5 years of historical data were needed, were not included if they were affiliated to a student health insurance over this period because data from this health insurance scheme are available only since 2015. This may lead to a selection bias and an overestimation of the vaccination coverage. It has been indeed demonstrated that higher income and education level or a better occupational category are associated with higher vaccination coverage rates (30).

Finally, in some health centers, individuals’ health care consumptions are not recorded and are therefore not available in SNDS database. As a result, some vaccinations may have been missed in this study. This could have led to an underestimation of the vaccination coverage.

In addition, the SNDS database does not make it possible to link the mother and the father of one child. It could happen that one of the two parents is vaccinated while the other is not, while the main principle of the cocooning strategy is to create a cocoon against pertussis around the infant. It is important that both parents are vaccinated to offer the optimal protection to a newborn in its first months of life.

## Conclusion

This study provides recent and valid data on pertussis vaccination coverage using a medico-administrative database, in a country with near-universal healthcare coverage and a unified national healthcare data system. To optimize the protection in infants, which is the main objective of the cocooning strategy, pertussis immunization coverage of adults and seniors needs to be improved. Moreover, cocooning vaccination linked to a parental project needs to be performed earlier, during pregnancy (for those around the mother) or as soon as possible in immediate *post-partum*, for instance during the maternity stay. A series of different and coordinated interventions need to be considered to protect newborns from pertussis. Indeed, GPs have a key role in the vaccination against pertussis, but a better sensibilization of other healthcare professionals, specifically those dedicated to pregnancy follow-up, is needed to develop the cocooning strategy, before and during the pregnancy, as well as in postpartum.

Now that the French National Authority of Health has recommended pertussis vaccination for pregnant women, it will be of interest to follow-up on the pertussis vaccination status of mothers and fathers over the next years.

## Data availability statement

The datasets presented in this article are not readily available due to NHS and SNDS rules, no data sharing is possible as access to data is restricted to habilitated and qualified researchers. Requests to access the datasets should be directed to the corresponding author.

## Ethics statement

The studies involving human participants were reviewed and approved by Ethics and Scientific Committee for Health Research, Studies and Evaluations (CESREES). Written informed consent from the participants’ legal guardian/next of kin was not required to participate in this study in accordance with the national legislation and the institutional requirements.

## Author contributions

CM and RV provided the supervision, conceived and designed the study, interpreted the data, and drafted the manuscript. NG and RC were members of the Scientific Committee and interpreted the data. FJ carried out the primary statistical analysis, including figures and table. MB and ML contributed to study design and interpretation of data. CM was the guarantor of the content of the manuscript. All authors critically reviewed and approved the final draft of the manuscript.
